# Neonatal Air Transport of Thoraco-Omphalopagus Conjoined Twins: A Case of Adaptation and Multidisciplinary Coordination

**DOI:** 10.3390/children12040423

**Published:** 2025-03-28

**Authors:** Bogdan Oprita, Teodor Nicolae Berea, Ruxandra Oprita

**Affiliations:** 1 Emergency Department, Floreasca Emergency Hospital, 014461 Bucharest, Romania; bogdan.oprita@umfcd.ro; 2 Faculty of Medicine, “Carol Davila” University of Medicine and Pharmacy, 020021 Bucharest, Romania

**Keywords:** neonatal transport, thoraco-omphalopagus conjoined twins, mechanical ventilation, helicopter transport, prenatal diagnosis

## Abstract

**Background**: The emergency transport of thoraco-omphalopagus conjoined twins is a rare occurrence in the medical field of emergency medicine with no specific guidelines on which medical personnel can rely upon. We present to you the case of the transport of thoraco-omphalopagus conjoined twins. **Methods:** Important changes were made to the configuration of the aircraft as to provide adequate ventilation (one twin ventilated manually with an ABV while the other benefiting from mechanical ventilation) and hemodynamic stability. **Results:** The team successfully orchestrated the transfer with no complications arriving during the transfer with the twins benefiting from specialized care in the intended tertiary care center. **Conclusions**: Our paper highlights the importance for innovative approaches in adapting neonatal transport equipment, including ventilation and monitoring for two critically ill patients in a space designed for one, all that underscores the importance of tailored neonatal transport solutions.

## 1. Introduction

Conjoined twins (CTs) are a rare embryological accident whose etiology is not fully understood. Recent studies show a prevalence that ranges from 1.02 to 1.34 per 100,000 births in Western populations [1,2,3] to 3.27 per 100,000 births in China [4], and with a dominance of the female sex at a 3:1 ratio [5]. The most common form of conjoined twins is thoracopagus, accounting for approximately 34% of cases [6]. Up to 90% of these cases present with a common pericardial sac, and with two- thirds presenting with a conjoined heart [7].

A precise diagnosis is required as this condition comes with a high risk of perinatal death due to congenital defects that are noncompatible with life, with death percentages rising to 70% in the first 48 h after delivery [8].

In this article, we shall report a case of conjoined thoraco-omphalopagus diagnosed at birth by caesarian section in a patient that did not undergo any prenatal screening and their urgent transportation to a major hospital due to their critical condition.

## 2. Case Report

On the 22nd of December 2015, an urgent request was made for the air transport of thoraco-omphalopagus conjoined twins (referred to as A and B), born earlier that day at the Constanta Emergency Clinical County Hospital. The transfer was intended for the “Marie Curie” Emergency Clinical Pediatric Hospital in Bucharest as this hospital was the most suited for the immediate care of these patients.

The 24-year-old mother G6P1 had not undergone antenatal care. The birth occurred prematurely at 33 weeks of gestation via cesarean section due to signs of fetal distress.

The twins were hypotrophic with a combined birth weight of 3000 g and were delivered by cesarean, in critical condition, at the Constanta Emergency Clinical County Hospital. Upon birth, both neonates presented with cyanosis, absent spontaneous respiration, weak gasping efforts, and barely perceptible heart activity with APGAR scores at 1 min of 2 for patient A and 3 for patient B.

Resuscitation efforts were promptly started. Both neonates were orotracheally intubated and placed on mechanical ventilation with intermittent positive pressure ventilation (IPPV) mode, FiO_2_ = 90%, PEEP = 5 cm H_2_O, respiratory rate (RR) = 55 breaths per minute, and inspiratory time (TI) = 0.45 s. The APGAR scores at 5 min were 3 for patient A and 4 for patient B. At 10 min, the neonates did not exhibit any cyanosis but were still not responding to stimuli and their heartrates were <100, which translates to an APGAR score at 10 min of 5 for both patients.

The transport team that was on site due to an earlier transport of a neonatal patient towards Constanta Hospital promptly came to aid to assure their transport was as swift as possible while providing the best care nonetheless. The aerial distance between the two cities is 207.75 km. The transfer time was estimated to be 45 min not including loading and unloading, nor take off and landing.

**Figure 1:**
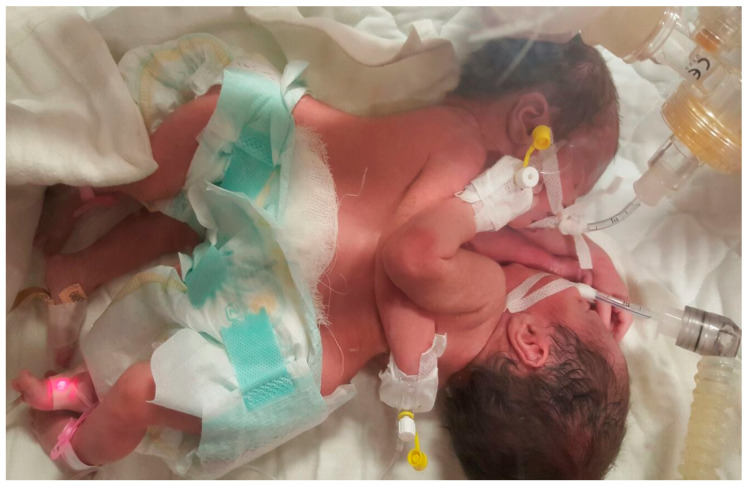
Thoraco-omphalopagus twins A and B upon initial examination by the transporting team.

Upon examination by the transport team (Figure 1), the twins were in a profoundly critical state, sharing a common thoraco-abdominal wall and a shared, dressed omphalocele. Both were intubated and mechanically ventilated. They exhibited hypotonia and hypoactivity. Twin A had an SpO_2_ of approximately 80% (despite FiO_2_ = 100%), while Twin B had an SpO_2_ of 90%. Cardiac sounds were faint in both. Both twins had peripheral venous catheters for intravenous fluid and electrolyte rebalancing, as well as nutritional support. A congenital cardiac anomaly was suspected, though no advanced imaging investigations were readily available. No laboratory data were provided.

**Figure 2:**
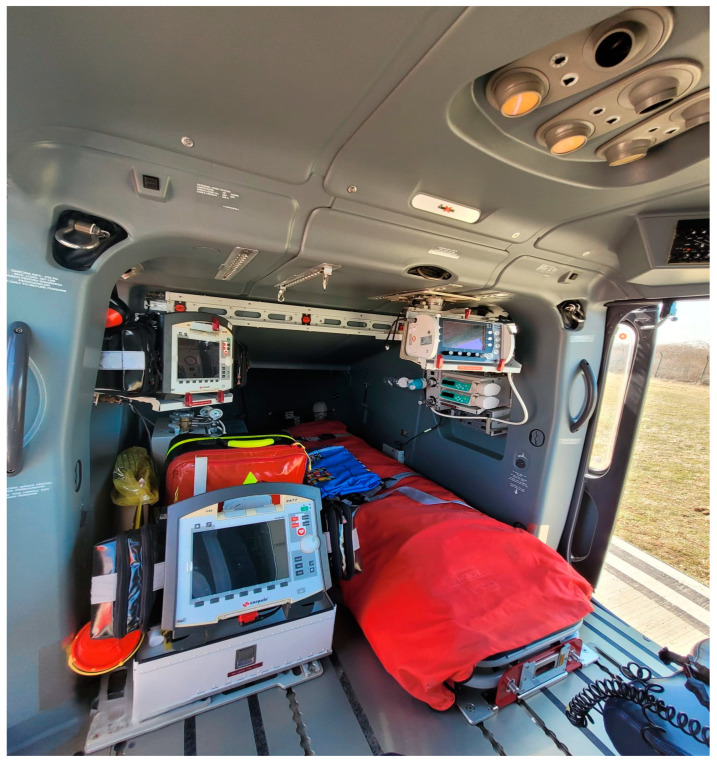
Initial configuration of the aircraft before the modifications.

The helicopter that was on site was an Airbus EC 135 (Airbus Helicopters, Marignane, France) (Figure 2) which required adaptation in order to receive the newborns for transport. The patients were transported in an Atom Transcapsule V-707 Intubator (Atom Medical International, Inc., Abbiategrasso, Italy).

**Figure 3:**
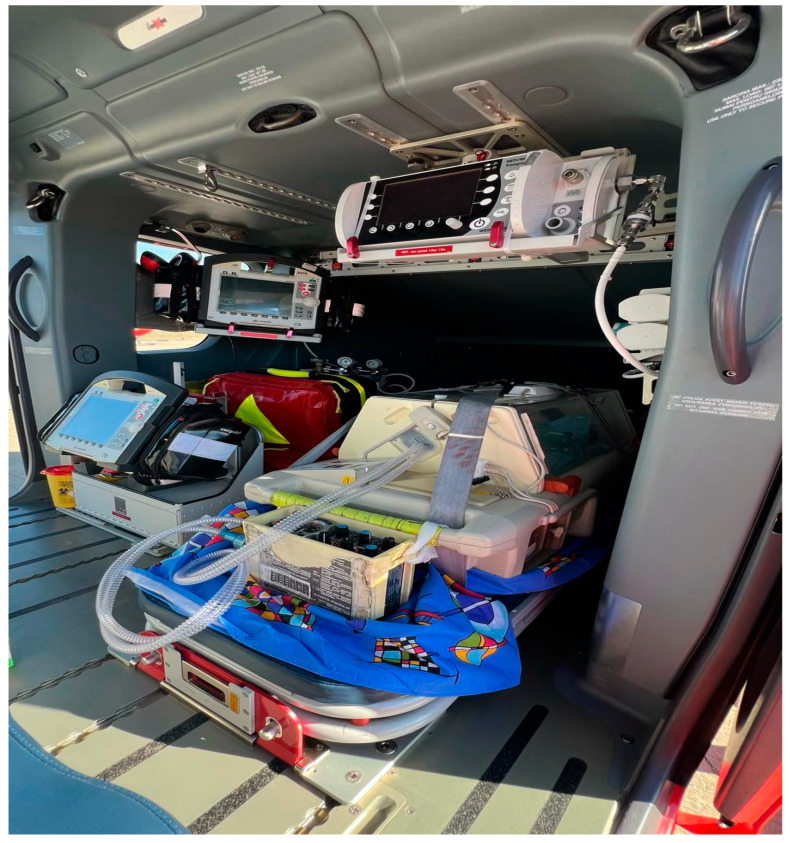
Modified cabin of the aircraft.

The assistant’s seat was repositioned facing forward, enabling twin A to be ventilated with the neonatal ventilator baby-PAC 100 (Smiths Medical MD, Inc., Minneapolis, MN, USA), and his vitals were monitored using a Corpuls 3 scope (Corpuls, Kaufering, Germany), while twin B was manually ventilated with a Ruben bag (Ambu, Copenhagen, Denmark) with additional oxygen flow, himself benefiting from continuous vital sign surveillance by the defibrillator monitor (Figure 3).

Twin A, in a more critical condition, was connected to the transport ventilator and ventilated using CMV + PEEP mode (RR = 60/min, FiO_2_ = 100%), while Twin B was ventilated through an endotracheal tube (ETT) with a neonatal resuscitation bag, supplemented with 3 L/min of oxygen, at a rate of approximately 60 breaths per minute. During the 90 min transport, twin A maintained an SpO_2_ between 78 and 85% and a heart rate (HR) between 145 and 160 bpm, while twin B had an SpO_2_ between 88 and 94% and an HR between 150 and 165 bpm (as measured by the transport pulse oximeter). The medical team, consisting of a physician and an assistant, divided responsibilities: one continuously manually ventilated twin B, while the other secured the incubator, connected the ventilator and oxygen sources, and ensured the stability of the transport monitor, ventilator, pulse oximeter, and infusion pumps.

In addition to maintaining the patients’ thermal stability, continuous monitoring, and intravenous rebalancing therapy, the most significant challenge in this case was providing mechanical ventilation to both patients within the confined space of the helicopter and incubator, with a high risk of accidental extubation.

Upon arrival in Bucharest, the twins were handed over to the neonatal transport team from Floreasca Emergency Hospital for transfer to Marie Curie Hospital, the transfer being made in an ambulance that was modified similarly to the aircraft.

## 3. Discussion

**Figure 4:**
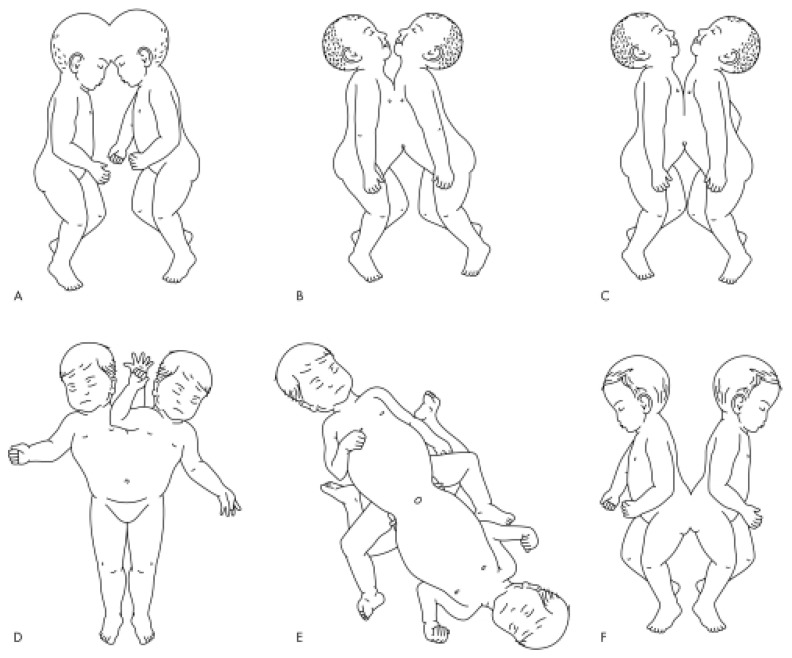
Common forms of conjoined twins: (**A**) craniopagus, (**B**) thoracopagus, (**C**) omphalopagus, (**D**) parapagus, (**E**) ischiopagus, and (**F**) pyopagus [14].

There are three categories of conjoined twins: twins with anterior (ventral) union, twins with dorsal (posterior) union, and those with lateral union [9,10]. Among these, depending of the site of the conjunction, they are further classified as cephalopagus (skull), thoracopagus (thorax), omphalopagus (abdomen), ischiopagus (pelvis), pygopagus (sacrum), and rachipagus (back) (Figure 4) [10]. Anterior (ventral) and/or abdomen fusion (referred to as thoraco-omphalopagus) is the most common form observed, with the fetal chest being the most involved, that altogether constitutes two-thirds of the cases [1,11].

Omphalo-thoracopagus twins have, generally, a poor prognosis due to a higher incidence of organ anomalies (e.g., cardiac, hepatic, biliary, limb defects, of the abdominal wall) [12,13]. Cardiac fusion is common (75%) and usually involves complex cardiac defects like common atrial mass, common right or left ventricles, two atrioventricular valves overriding a complex ventricular structure, shared pericardium (90% of thoraco-omphalopagus twins), and also defects affecting the major heart vessels (aorta, pulmonary veins) [14,15]. All of this is important as it directly affects the prognosis of the separation surgery that historically has been poor and marked by numerous complications, especially for those conjoined at multiple anatomic regions. In one study of 40 cases of conjoined twins, the survivability of those that underwent surgery was reported at 60% [16], while another study from the UK and South Africa reported a total survival rate of 66% [17].

There is a notable scarcity of detailed information concerning the safe transport of conjoined twins, particularly in critical conditions requiring specialized care. This raises a critical question: how should we proceed when faced with conjoined twins from an unmonitored pregnancy, in critical condition, and requiring transfer from a healthcare facility unequipped to manage such a complex case?

Managing respiratory function is a major challenge in thoraco-omphalopagus conjoined twins. These twins often experience mechanical respiratory difficulties due to smaller or fused thoracic cavities, possibly with shared diaphragms. Each twin typically has independent respiratory centers, leading to asynchronous respiratory rhythms. Paradoxical chest movements are commonly observed, particularly in those with extensive thoracic fusion. This respiratory asynchrony is exacerbated during periods of agitation, impairing gas exchange, as reflected in decreased peripherical oxygenation values. Effective secretion clearance from the bronchial tree is also more efficient when coughing is synchronized between the twins. In cases of respiratory insufficiency, clinical assessment, along with gas analysis and imaging, plays a vital role in determining respiratory intervention strategies.

A potential alternative to the ventilation strategy employed could have involved using two transport ventilators or a Y-piece connector to ventilate both twins with a single ventilator, although this might result in insufficient tidal volumes due to reduced thoracic expansion, making asynchronous ventilation preferable for optimizing gas exchange.

A thorough examination was performed upon arrival in the tertiary care hospital’s NICU. After CT and MRI scans, it was observed that the twins were fused at the lower thoracic and abdominal levels, sharing a conjoined heart (with twin B’s left ventricle in continuity with twin A’s right ventricle), a common liver, and a shared omphalocele, though each had separate lungs, stomachs, gallbladders, spleens, kidneys, and urinary tracts.

Even though the initial suspicion of a cardiac anomaly was confirmed, its extent was not a severe as initially thought; there was already a high chance that the patients had two separate left atriums and two separate left ventricles, because upon continuous monitoring of the twins, we observed two distinct heart rates. Electrocardiograms were performed upon arrival that showed distinct sinus heart rhythms without conduction abnormalities. The presence of a single QRS complex suggests that separation may not be feasible, whereas two distinct ECG patterns do not guarantee successful separation in cases of cardiac conjunction. The twins were stabilized while waiting further surgical evaluation.

The particularities of this case include the rarity of such births, the absence of prenatal monitoring despite current advanced prenatal surveillance methods, and the simultaneous air transport of two critically ill, mechanically ventilated neonates using equipment designed for a single patient.

## 4. Conclusions

This case highlights the logistical and medical complexities involved in the transport of critically ill thoraco-omphalopagus conjoined twins. The need for innovative approaches in adapting neonatal transport equipment, including ventilation and monitoring for two critically ill patients in a space designed for one, underscores the importance of tailored neonatal transport solutions.

The rapid adaptation of the aircraft in order to successfully assure the transfer of the twins was a crucial step for improving their overall survival chances in the long term. Although the transport was without incident, the monitorization of the vitals of these patients requires further study so as to avoid improvisations, as was our case; this raises the question of an adapted ventilator that can assure higher tidal volumes that could suffice for two neonatal patients at the same time.

## Data Availability

The original contributions presented in the study are included in the article; further inquiries can be directed to the corresponding authors.
